# Fusobacterium nucleatum promotes anastomotic leakage by activating epithelial cells through the NOD1/RIPK2/ERK signalling pathway to drive IL‐1β‐induced neutrophil chemotaxis and collagen degradation

**DOI:** 10.1002/ctm2.70262

**Published:** 2025-03-05

**Authors:** Zixian Wei, Liqing Bao, Bowen Li, Jinhua Yang, Jijiao Wang, Fangqi Xu, Hubin Wenren, Kunyu Zhang, Shang Shi, Liying Yan, Xin Tao, Tianqi Zhang, Zhiyue Wang, Yang Liu

**Affiliations:** ^1^ Department of Hepatobiliary and Pancreatic Surgery Ningbo No. 2 Hospital Ningbo Zhejiang China; ^2^ Department of Hepatobiliary and Pancreatic Surgery The First Affiliated Hospital of Harbin Medical University Harbin Heilongjiang China; ^3^ Ningbo Key Laboratory of Intestinal Microecology and Human Major Diseases Ningbo China; ^4^ Department of Breast Surgery Ningbo Medical Center Lihuili Hospital Ningbo China

1

Dear Editor:

We herein suggest that *Fusobacterium nucleatum* (*F. nucleatum*) disrupts anastomotic healing. It promotes the movement and activation of neutrophils, which increases MMPs production. These MMPs break down collagen, weakening the anastomosis and causing leakage.

Anastomotic leakage (AL) is a critical and potentially fatal complication that may arise following colorectal surgery.^1^, ^2^ Despite its clinical significance, no specific risk factors or surgical techniques have been identified that can effectively improve anastomotic healing.^3^ Recent studies indicate a possible connection between gut microbiota imbalances and the occurrence of AL.^4^, ^5^, ^6^ We previously demonstrated an increased abundance of *F. nucleatum* in patients who developed AL.^7^ Inflammation has been implicated in anastomotic leakage (AL), with neutrophils (NEs) being the main inflammatory cells involved in acute colitis.^8^ Matrix metalloproteinases (MMPs) play a crucial role in tissue damage associated with AL.^9^ Neutrophils contribute to tissue breakdown in AL by releasing MMPs.^10^ This study aims to investigate the impact of *F. nucleatum* on AL, with a particular emphasis on the role of neutrophils in this process.

To establish that *F. nucleatum* colonisation induces AL, we developed a colon anastomotic healing model in germ‐free rederivation mice. Mice were inoculated via enema with *F. nucleatum*, *E. coli*, or PBS from the day of surgery (POD0) to POD3. All mice were sacrificed on POD7, and a laparotomy was performed to assess the gross anastomotic healing (Figure 1A). *F. nucleatum* colonised both mucosal and submucosal layers (Figure 1B and C), leading to poor anastomotic healing, as evidenced by leakage, peritoneal contamination, and visible dehiscence (Figure 1D). The anastomotic healing scores were higher (poorer healing) in the *F. nucleatum* group, with increased inflammatory cell infiltration (Figure 1E) and weak collagen deposition (Figure 1F). Neutrophil activation and enhanced MMP9 deposition were observed (Figure 1G), with gelatin zymography showing increased MMP9 and NGAL‐MMP9 (Figures 1H and J). Collagenase activity was also elevated (Figure 1I), and multiplex immunofluorescence revealed co‐localisation of *F. nucleatum*, MMP9, and neutrophil markers, linked to reduced collagen deposition (Figure 1K). All these suggest that *F. nucleatum* contribute to neutrophil activation and collagen degradation contribute to AL.

**FIGURE 1:**
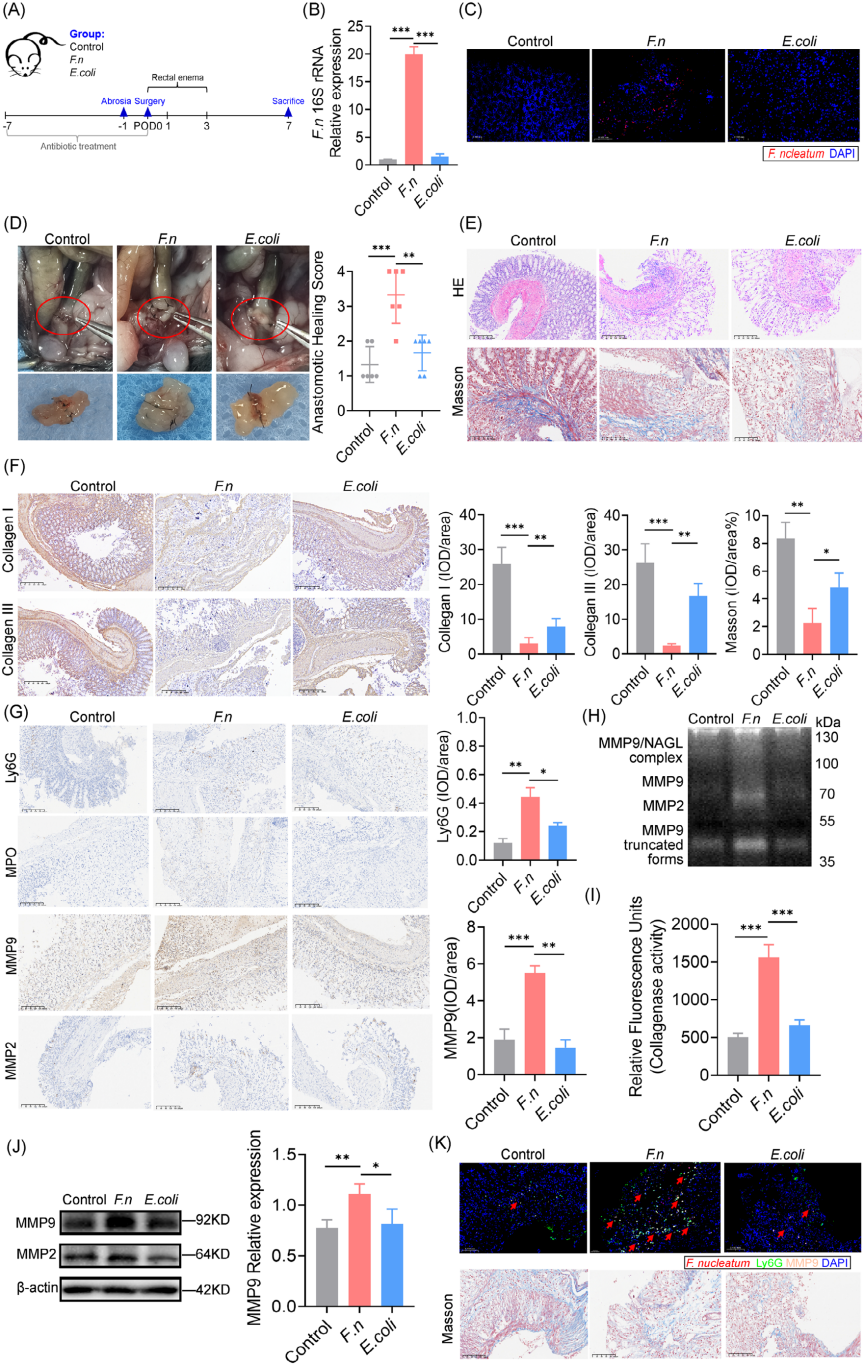
F. nucleatum promotes neutrophil chemotaxis, increasing MMP9 secretion and aggravating anastomotic leakage. (A) Schematic representation of the anastomosis model. Mice were randomly assigned to one of three experimental groups: Control, *F. nucleatum* (F.n), and *E. coli* (E.c) groups. (B) Quantification of *F. nucleatum* 16S rRNA gene expression in anastomotic tissues by RT‐qPCR (*n* = 6 mice per group). Data are presented as relative expression normalised to control. (C) Representative fluorescence in situ hybridisation (FISH) images showing *F. nucleatum* 16S rRNA (red) in mouse anastomotic tissues. Nuclei were counterstained with DAPI (blue). Objective lens, 20×; scale bar = 100 µm. (D) Macroscopic assessment of anastomotic healing and corresponding anastomotic healing scores (AHS) in the three experimental groups (*n* = 6 mice per group).(E) Haematoxylin and eosin (HE) and Masson's trichrome staining of anastomotic tissues from each group, showing histopathological features of healing and fibrosis (*n* = 6 mice per group). (F) Immunohistochemistry (IHC) staining for collagen I and collagen III in anastomotic tissues from the control, *F. nucleatum*, and *E. coli* groups (*n* = 6 mice per group). (G) Representative IHC staining for Ly6G, MPO, and MMP9 in anastomotic tissues from the three groups, demonstrating neutrophil infiltration and MMP9 expression (*n* = 6 mice per group). (H) Gelatin zymography showing matrix metalloproteinase (MMP) activity in anastomotic tissue lysates. (I) Collagenase activity in anastomotic tissues from the three experimental groups, assessed by enzymatic activity assay. (J) Immunoblot analysis of MMP9 and MMP2 expression in protein extracts from anastomotic tissues. (K) Immunofluorescence co‐staining for *F. nucleatum* 16S rRNA (red), MMP9 (pink), and Ly6G (green) in anastomotic tissues, with Masson's trichrome staining of the same tissue sections for collagen deposition. *p < .05, **p < .01, ***p < .001. All data are presented as the means ± SD.

To evaluate the direct influence of *F. nucleatum* on neutrophils, we infected neutrophils with *F. nucleatum* in vitro. This led to neutrophil activation, as evidenced by an increase in reactive oxygen species (ROS) production (Figure S1F). The MMPs activity in neutrophil culture supernatants, including NGAL‐MMP9 complexes, was significantly elevated (Figure S1A). Furthermore, the collagenase activity in the supernatant was markedly higher in the F. nucleatum‐infected group (Figure S1C). Additionally, F. nucleatum stimulated MMP9 and MMP2 expression and secretion from neutrophils (Figure S1B and D). These findings indicated that F. nucleatum causes neutrophil chemotaxis and activation, leading to MMP‐mediated collagen degradation.

To investigate the effect of neutrophils in F. nucleatum related AL, neutrophil depletion was achieved using an anti‐Ly6G antibody (Figure 2A) and confirmed by reduced neutrophil counts and spleen infiltration (Figure S2A and B). Neutrophil depletion mitigated *F. nucleatum*’s effects, restoring healing with lower anastomotic healing scores (Figure 2B). Collagen formation, assessed by Masson's trichrome staining and IHC, was restored, while *F. nucleatum*‐induced neutrophil chemotaxis, activation, and MMP9 deposition were reduced (Figure 2C). Gelatin zymography showed diminished MMP2, MMP9, and NGAL‐MMP9 activity in neutrophil‐depleted tissues (Figure 2E), with suppressed expression of MMP2 and MMP9 (Figure 2D). Collagenase activity was also significantly reduced (Figure 2F). These findings suggest that neutrophils promote AL by secreting MMPs, leading to collagen degradation.

**FIGURE 2:**
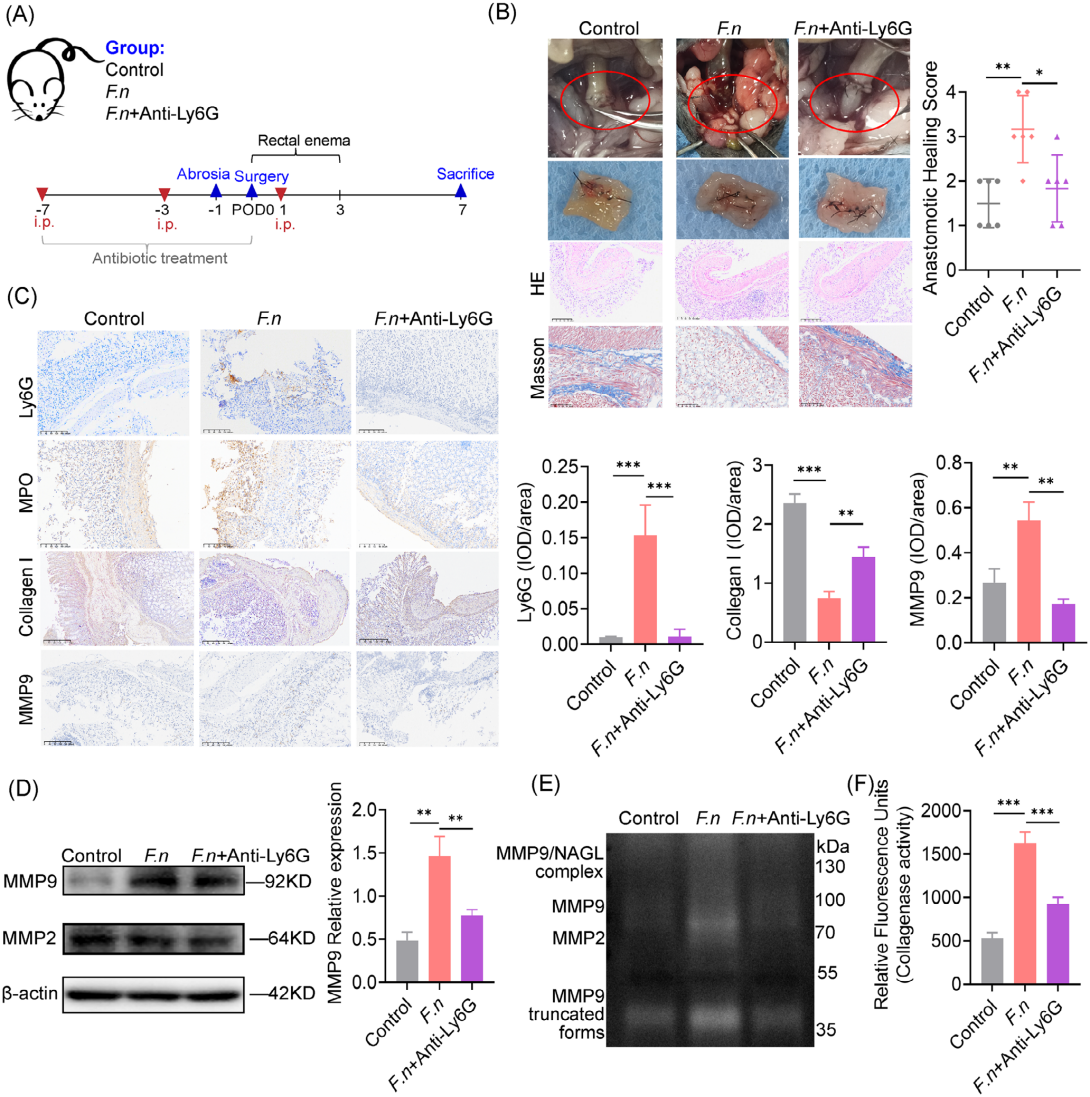
Collagen degradation and tissue collagenase activity were decreased in mouse anastomotic tissue when neutrophils were reduced. (A) Diagram of the mouse anastomosis model. Mice were randomly assigned to three groups: PBS control, *F. nucleatum*, or *F. nucleatum* + anti‐Ly6G antibody.(B) Representative macroscopic images of anastomotic healing and corresponding anastomotic healing scores (AHS) for the three groups (*n* = 6 mice per group). Haematoxylin and eosin (HE) and Masson's trichrome staining of anastomotic tissues were performed.(C) Immunohistochemistry (IHC) staining for Ly6G, myeloperoxidase (MPO), collagen I, and MMP9 in anastomotic tissues from all groups (*n* = 6 mice per group).(D) Western blot analysis of MMP9 and MMP2 in tissue extracts from anastomotic sites (*n* = 6 mice per group). (E) Gelatin zymography to assess the activity of matrix metalloproteinases (MMPs) in anastomotic tissue lysates (*n* = 6 mice per group).(F) Collagenase activity in anastomotic tissue, assessed by enzymatic activity assay. *p < .05, **p < .01, ***p < .001. All in vitro experiments were repeated three times. All data are presented as the mean ± SD.

Since IECs produce neutrophil chemokines during inflammation, we hypothesised that F. nucleatum infection stimulates their secretion, driving neutrophil infiltration. Co‐culture of Caco‐2 cells with F. nucleatum showed direct adherence (Figure S3), and transcriptomic analysis revealed upregulation of 1254 genes, including IL‐1β and IL‐8 (Figure 3A). GO enrichment analysis revealed the activation of pathways associated with neutrophil chemotaxis (Figure 3B). qPCR confirmed increased IL‐1β and IL‐8 expression (Figure 3C and D). Meanwhile, ELISA results showed an increase in IL‐1β concentration in the culture medium (Figure 3E). Negligible IL‐1β secretion was observed in F. nucleatum‐only cultures, confirming Caco‐2 cells as the source (Figure 3F). These results suggest that F. nucleatum induces neutrophil chemotaxis by promoting IL‐1β secretion from IECs.

**FIGURE 3:**
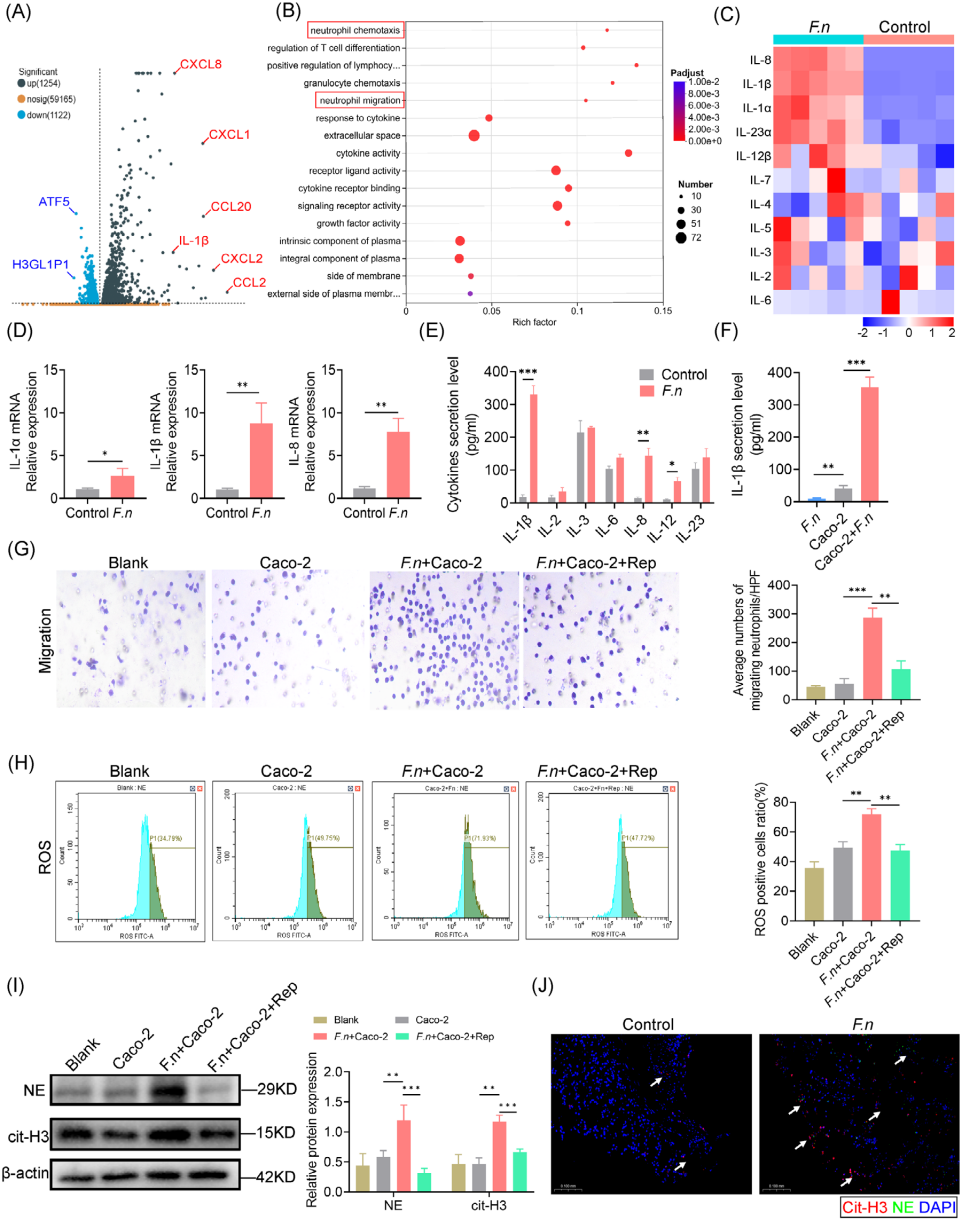
*F. nucleatum* interacts with intestinal epithelial cells and induces their IL‐1β secretion in vitro. (A) Volcano plot showing gene expression changes in Caco‐2 cells co‐cultured with *F. nucleatum*. (B) Gene Ontology (GO) enrichment analysis of differentially expressed genes in Caco‐2 cells induced by *F. nucleatum* co‐culture. (C) Heatmap illustrating changes in the expression of major interleukin genes in Caco‐2 cells upon *F. nucleatum* co‐culture. (D) RT‐qPCR analysis of mRNA levels for IL‐1α, IL‐1β, and IL‐8 in Caco‐2 cells induced by *F. nucleatum*. (E) ELISA assessment of cytokine secretion in Caco‐2 cells induced by *F. nucleatum*. (F) ELISA measurement of IL‐1β secretion in Caco‐2 cells. (G) Representative images and quantification of neutrophil migration in a Transwell system with an 8‐micron pore size. (H) Representative flow cytometry (FACS) plots for reactive oxygen species (ROS) staining in neutrophils, and quantification of ROS‐positive cell ratios. (I) Immunoblot analysis of neutrophil elastase (NE) and citrullinated histone H3 (Cit‐H3) in neutrophil extracts. (J) Representative immunofluorescence images showing Cit‐H3 (red), neutrophil elastase (green), and DAPI (blue) localisation in anastomotic tissues (objective lens 20×; scale bar = 100 µm). *p < .05, **p < .01, ***p < .001. All in vitro experiments were repeated three times. All data are presented as the mean ± SD.

To investigate whether *F. nucleatum*‐infected IECs can directly induce neutrophil migration, we conducted in vitro Transwell assays and observed that *F. nucleatum*‐infected IECs promoted significant neutrophil migration. This was inhibited by the addition of an IL‐1β receptor blocker, supporting that *F. nucleatum*‐infected IECs mediate neutrophil chemotaxis through IL‐1β secretion (Figure 3G). Treatment with an IL‐1β receptor blocker also inhibited neutrophil activation, as evidenced by reduced ROS staining (Figure 3H).

Neutrophil extracellular traps are web‐like structures released by neutrophils, which can work as MMPs reservoirs to induce tissue remoulding. We observed that *F. nucleatum*‐infected Caco‐2 cells exhibited upregulation of neutrophil elastase, myeloperoxidase, and citrullinated histone H3, all of which are key components involved in the formation of web‐like structures that make up neutrophil extracellular traps (NETs) (Figure 3I). Immunofluorescence staining confirmed the spatial presence of NETs markers at the anastomotic site, indicating their accumulation and potential involvement in the local inflammatory response (Figure 3J).

Although the studies above demonstrated that *F. nucleatum* targeted IECs to induce neutrophil infiltration via IL‐1β, it was unclear how this was occurring. A KEGG enrichment analysis of the upregulated gene sets revealed the involvement of several inflammatory pathways, including the Nucleotide‐Binding Oligomerization Domain Containing (NOD), TLR and MAPK pathways (Figure 4A). qPCR showed that NOD1, but not NOD2 or TLR4, was significantly upregulated following infection (Figure 4B). NOD1 and Receptor Interacting Serine/Threonine Kinase 2 (RIPK2) protein expression was upregulated, with increased ERK phosphorylation (Figure 4C), confirmed by histochemical staining in mouse anastomosis tissue (Figure 4D). Silencing NOD1 reduced IL‐1β secretion, RIPK2 expression, and ERK phosphorylation, without altering bacterial invasion (Figure 4E–G). Inhibiting RIPK2 (WEHI‐345) or ERK (U0126) also decreased IL‐1β production (Figure 4I–K). These indicated that F. nucleatum induces IL‐1β secretion of IECs through the NOD1/RIPK2/ERK pathway.

**FIGURE 4:**
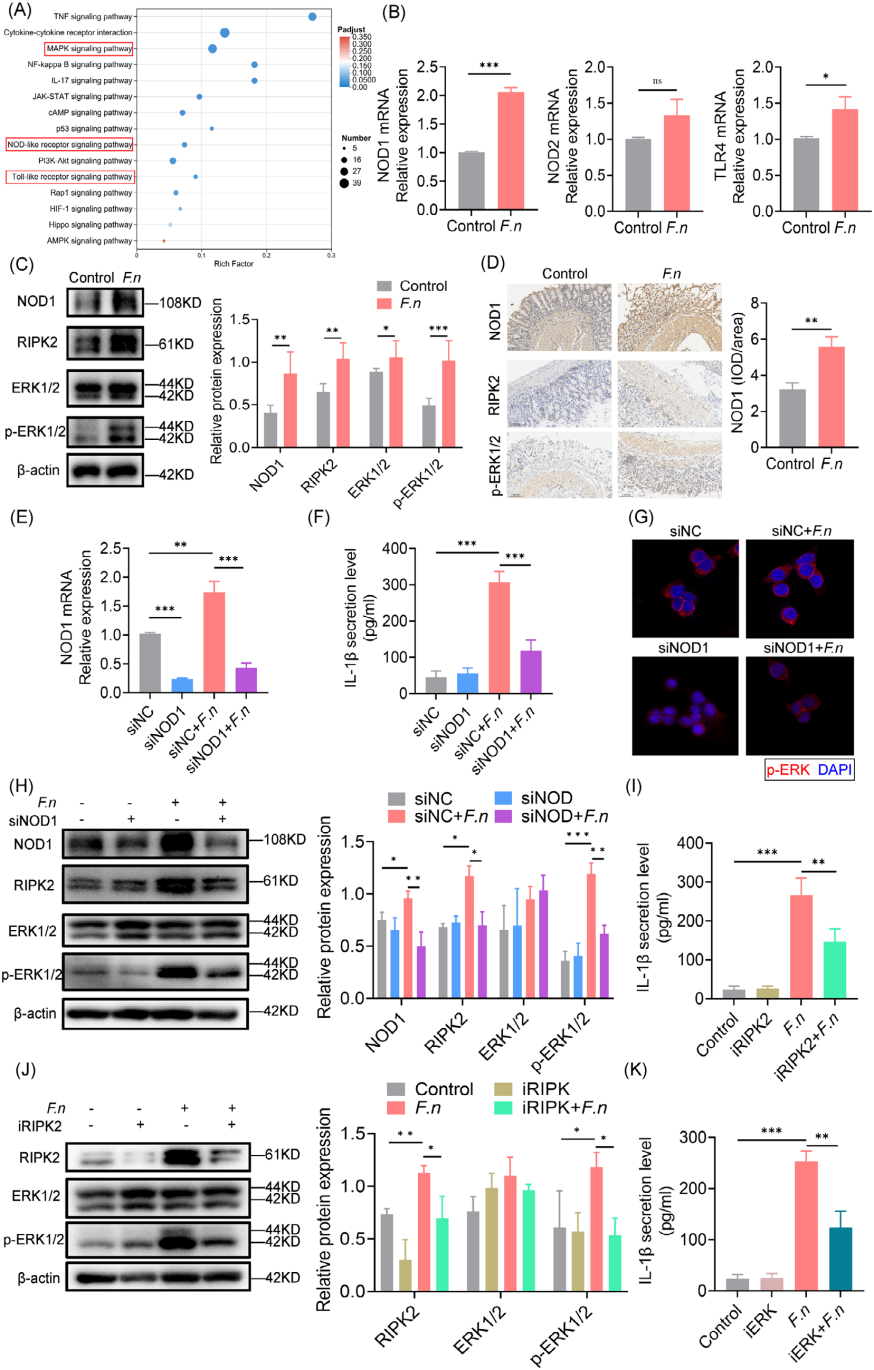
Exposure to *F. nucleatum* induces IL‐1β secretion by Caco‐2 cells via the NOD1/RIPK2/ERK signalling pathway. (A) KEGG pathway analysis showing altered gene expression in Caco‐2 cells induced by *F. nucleatum* infection. (B) mRNA expression levels of NOD1, NOD2, and TLR4 in Caco‐2 cells, assessed by RT‐qPCR. (C) Immunoblot analysis of protein extracts from Caco‐2 cells using the indicated antibodies. (D) IHC staining for NOD1, RIPK2, and p‐ERK1/2 in anastomotic tissues from control and *F. nucleatum* groups. (E) Suppression of NOD1 expression in Caco‐2 cells transfected with NOD1 siRNA, as determined by quantitative RT‐PCR. (F) IL‐1β secretion in Caco‐2 cells following NOD1 knockdown, assessed by ELISA. (G) Immunofluorescence staining of p‐ERK (red) and DAPI (blue) in NOD1‐knockdown Caco‐2 cells cultured with or without *F. nucleatum*. (H) Immunoblot analysis of protein extracts from Caco‐2 cells treated with NOD1 siRNA, using the specified antibodies. (I) IL‐1β secretion in Caco‐2 cells following treatment with a RIPK2 inhibitor, assessed by ELISA. (J) Immunoblot analysis of protein extracts from Caco‐2 cells following treatment with a RIPK2 inhibitor, using the specified antibodies. (K) IL‐1β secretion in Caco‐2 cells following treatment with an ERK inhibitor, assessed by ELISA. *p < .05, **p < .01, ***p < .001. All in vitro experiments were repeated three times. All data are presented as the mean ± SD.

In conclusion, this study revealed the negative impact of *F. nucleatum* on anastomotic healing. We demonstrated that *F. nucleatum* promotes the chemotaxis and activation of neutrophils and increases their secretion of MMPs, leading to collagen degradation and promoting anastomotic leakage.

## AUTHOR CONTRIBUTIONS

**Zixian Wei, Zhiyue Wang, Liqing Bao** and **Bowen Li**: Writing—original draft; investigation; methodology; conceptualisation; formal analysis and data curation. **Jinhua Yang, Jijiao Wang, Fangqi Xu, Hubin Wenren, Kunyu Zhang** and **Shang Shi**: Investigation; visualisation and software. **Liying Yan, Xin Tao** and **Tianqi Zhang**: Investigation. **Yang Liu**: Funding acquisition; project administration; resources; supervision; writing—review; validation. All authors read and approved the final version of the manuscript.

## CONFLICT OF INTEREST STATEMENT

No potential conflict of interest was reported by the authors.

## FUNDING INFORMATION

This research was supported by the Natural Science Foundation of China (Grant No. 82300631), Joint Funds of the National Natural Science Foundation of China (Grant No. U23A20458), Ningbo Top Medical and Health Research Program (Grant No. 2022010101), and Key Laboratory of Intestinal Microecology and Major Human Diseases in Ningbo (Grant No. 2023016).

## ETHICS STATEMENT

The experimental procedures were approved by the Ethics Review Committee of Guoke Ningbo Life Science and Health Industry Research Institute (GK‐2023‐XM‐0009 and GK‐2022‐12‐031) and were performed following Regulations for the Administration of Affairs Concerning Experimental Animals in Zhejiang Province.

## Supporting information



Supporting information

Supporting information

## Data Availability

Data for this study may be requested from the corresponding author where appropriate.
