# Supplementary material for: Fusobacterium nucleatum promotes anastomotic leakage by activating epithelial cells through the NOD1/RIPK2/ERK signalling pathway to drive IL‐1β‐induced neutrophil chemotaxis and collagen degradation
Source: Clin Transl Med. 2025 Mar 5;15(3):e70262. doi: 10.1002/ctm2.70262 (PMC12128141; doi:10.1002/ctm2.70262)
Supplement: Supplementary file 1 — Supporting information [file CTM2-15-e70262-s002.docx]

**Figure S1**

**
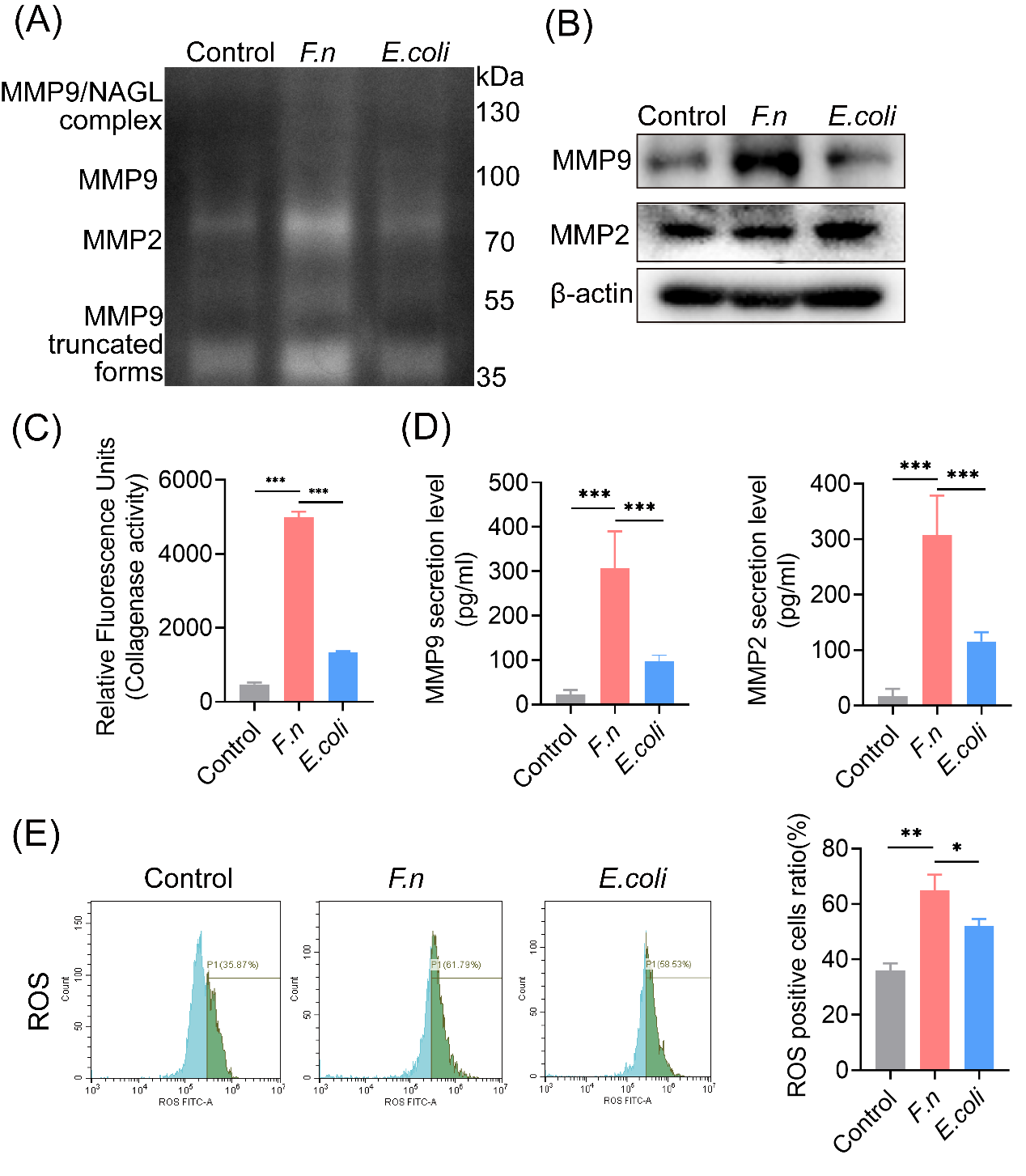
**

(A) Gelatin zymography showed the activity of MMPs of neutrophils after treatment in vitro.

(B) Immunoblot analysis of MMP9 and MMP2 of neutrophils after treatment in vitro.

(C) The activity of collagenase in vitro.

(D) The secretion of MMP9 and MMP2 of neutrophils after treatment in vitro.

(E) After treatment, representative FACS plots for ROS staining gated on neutrophils, the ROS-positive cell ratios are shown in vitro.

**Figure S2**

**
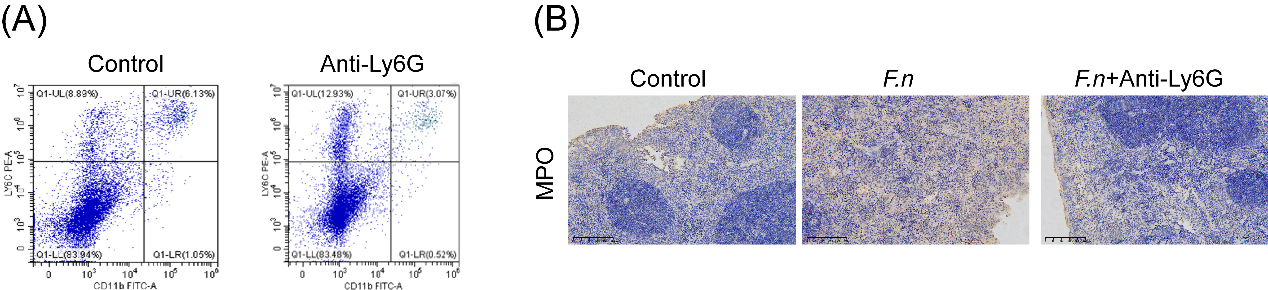
**

(A)Validation of neutrophil depletion of colon tissues by anti-Ly6G antibody by flow cytometry.

(B)Representative immunohistochemical staining showed the expression level of MPO in spleen tissues (objective lens, 10×), scale bar=200μm.

**Figure S3**


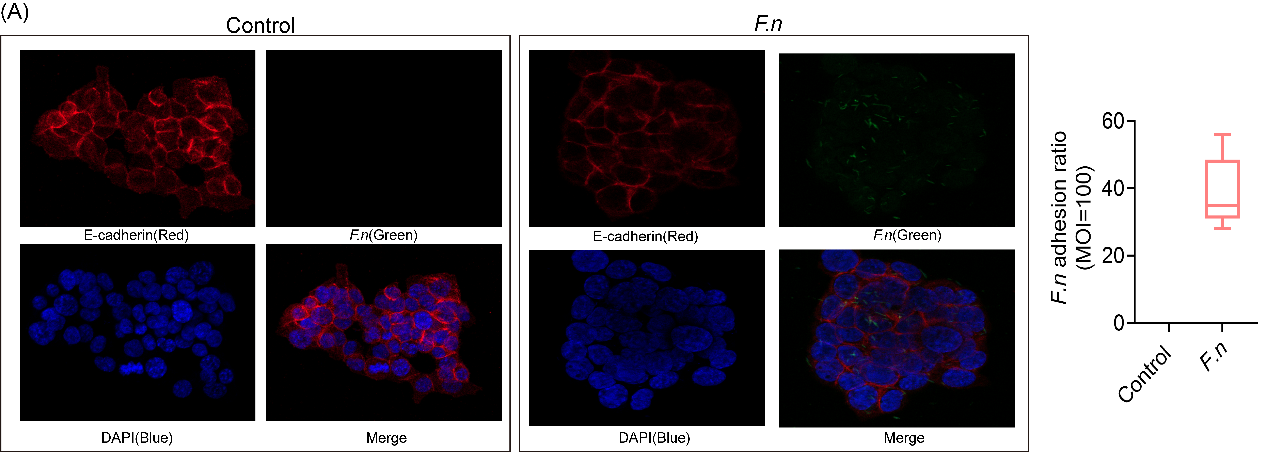


1. *F. nucleatum* adhesion to Caco-2 cells observed by confocal microscopy.

**Figure S4
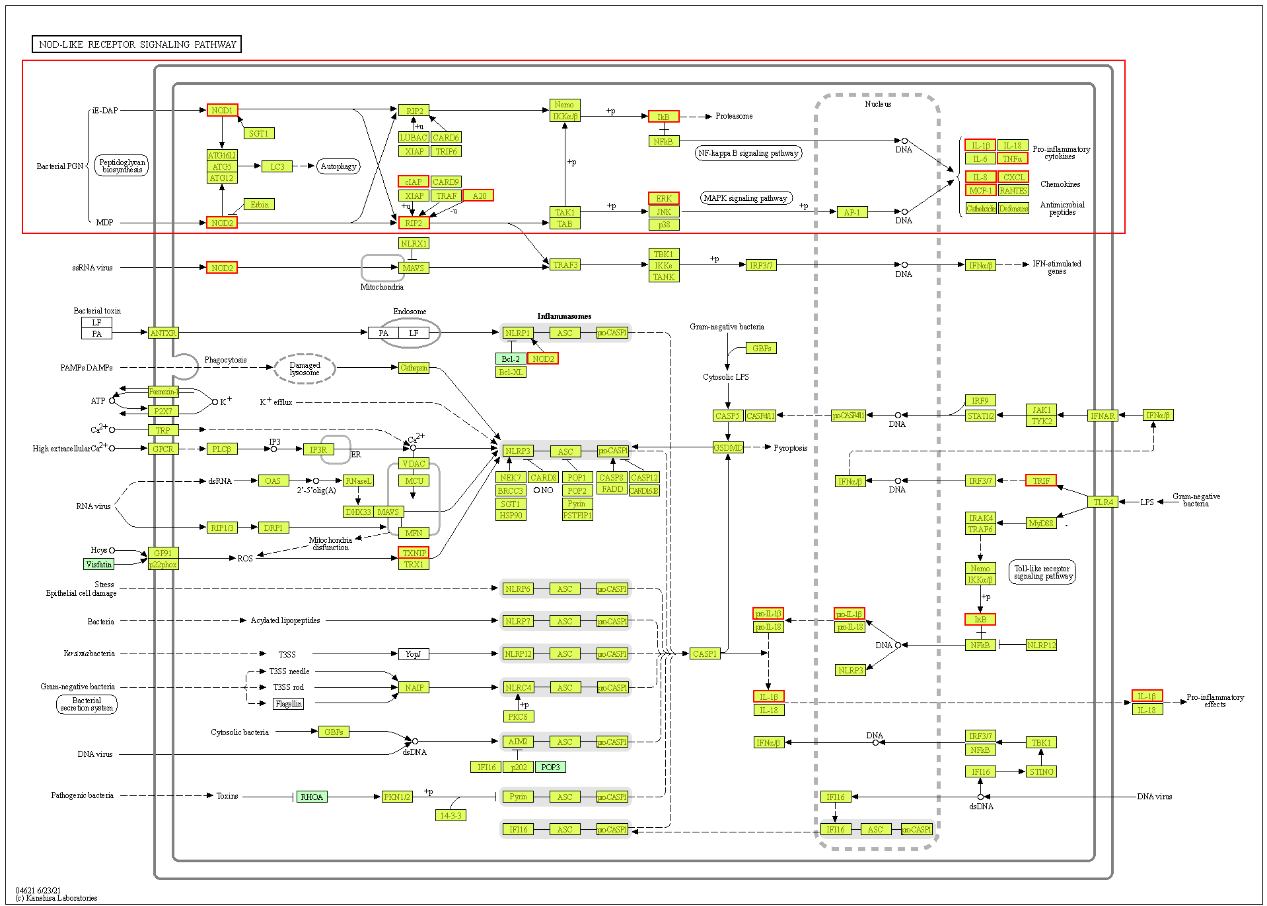
**

Nod-like receptor signaling pathway.

**Figure S5
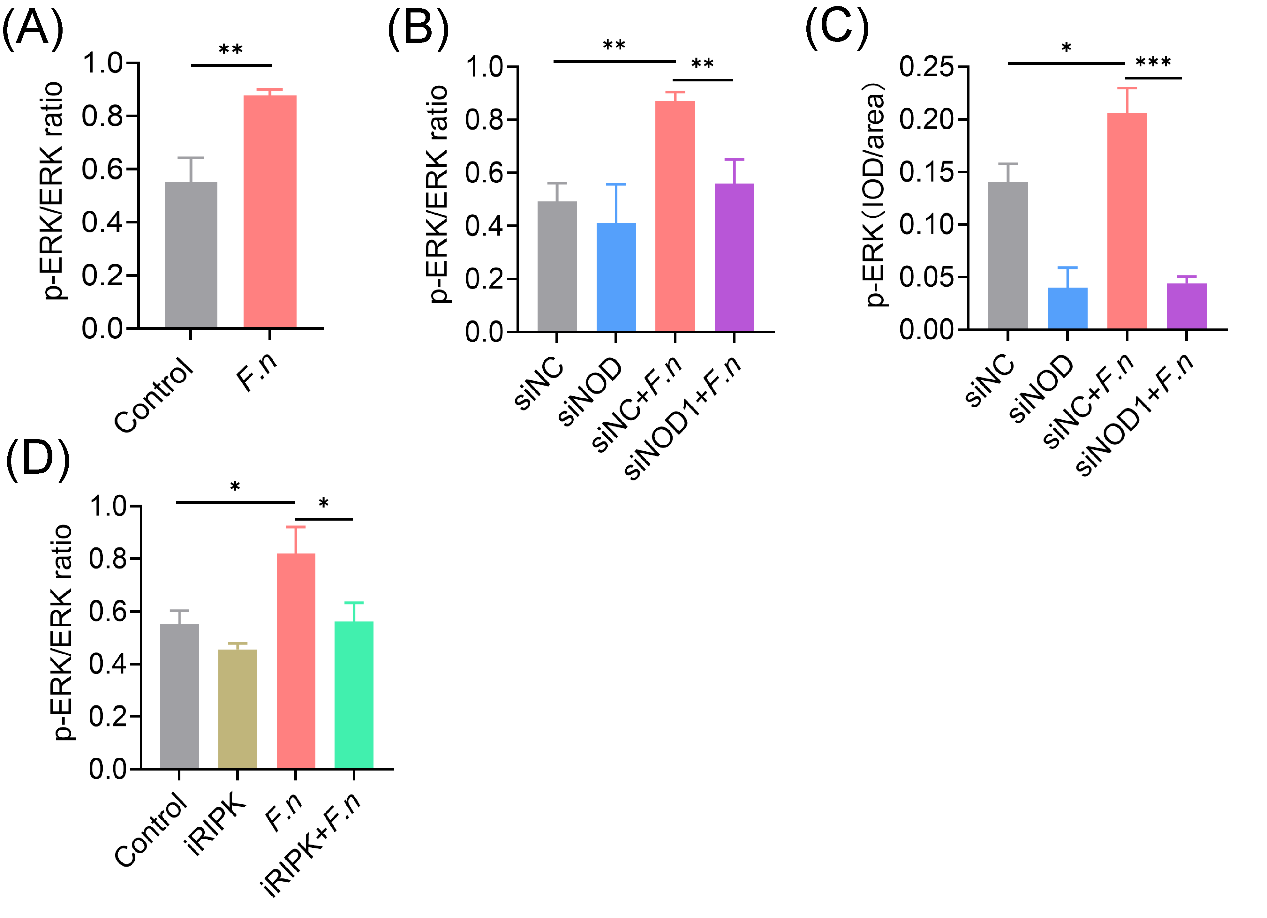
**

(A) Quantitative assessment of the ratio of p-ERK/ERK after co-cultured with *F. nucleatum*.

(B) Quantitative assessment of the ratio of p-ERK/ERK after applying siNOD1.

(C) Quantitative assessment of the phosphorylated ERK fluorescence intensity after applying siNOD1.

(D) Quantitative assessment of the ratio of p-ERK/ERK after applying WEHI-345.
