# Supplementary material for: Fusobacterium nucleatum promotes anastomotic leakage by activating epithelial cells through the NOD1/RIPK2/ERK signalling pathway to drive IL‐1β‐induced neutrophil chemotaxis and collagen degradation
Source: Clin Transl Med. 2025 Mar 5;15(3):e70262. doi: 10.1002/ctm2.70262 (PMC12128141; doi:10.1002/ctm2.70262)
Supplement: Supplementary file 2 — Supporting information [file CTM2-15-e70262-s001.docx]

**Method**

**1 Mouse model**

Six-week-old male C57BL/6J mice (20 to 22 g; Charles River; Beijing) were used in all experiments. Germ-free rederivation was achieved through antibiotic treatment, with antibiotics administered 1 week prior to surgery by adding ampicillin (0.2 mg/ml), neomycin (0.2 mg/ml), metronidazole (0.2 mg/ml), and vancomycin (0.1 mg/ml) to sterile drinking water.

Then mice were anesthetized through an intraperitoneal injection of tribromoethanol (Avertin 250 mg/kg). Following skin incision and intestine exposure, a cut was made across the sigmoid colon perpendicular to the direction of the colon and extending across 80-90% the width of the colon. An anastomosis was performed using 4 to 6 simple interrupted varus 8–0 proline sutures. Integrity was tested by distending the distal colon with saline via enema using a gavage needle. All mice were volume resuscitated with 1 ml of normal saline, then the abdomen was closed in 2 layers with 4–0 silk sutures. On postoperative day (POD) 1, mice were randomly assigned to the following treatment groups: phosphate-buffered solution (PBS) rectal enema (0.1 ml, PBS group), *F. nucleatum* rectal enema (1×10^8^ CFU *F. nucleatum* resuspended in 0.1 ml PBS, *F. nucleatum* group), and *E. coli* rectal enema (1×10^8^ CFU *E. coli* resuspended in 0.1 ml PBS, *E. coli* group). In all groups, the rectal enema was performed twice daily using a blunt tip gavage needle, and the enemas continued to be administered until postoperative day 3 (POD3). On POD7, all mice were euthanized, and anastomotic tissue samples were collected for further studies. To block neutrophil recruitment and activation, mice were injected intraperitoneally with 350 μg of anti-Ly6G (clone1A8, Bickel, Catlog# BE0075-1) antibodies on the first and fourth days. The animal experiment was repeated twice to demonstrate replicability.

The anastomotic healing score (AHS) was assessed using the following scale: 0, normal healing; 1, flimsy adhesions; 2, dense adhesions without abscess or intraperitoneal contamination; 3, dense adhesions with a gross abscess at the anastomotic site; and 4, gross leakage with peritoneal contamination and visible anastomotic dehiscence.

**2 Cell culture**

The human epithelial cell line, Caco-2, was purchased from the Shanghai Institute of Cell Biology (Shanghai, China) and maintained in DMEM with 20% fetal bovine serum (FBS) and 1% penicillin–streptomycin. The cells were cultured at 37°C in a humidified 5% CO_2_ atmosphere. To differentiate neutrophil-like cells, 1.25% DMSO was added to the culture medium after HL-60 cells were passaged and differentiated for three days. Differentiation was confirmed by flow cytometry, and the resulting neutrophil-like cells were used in subsequent experiments.

**3 Bacterial cultivations**

Fusobacterium nucleatum strain ATCC25586 (ATCC, Manassas, VA) was cultured overnight at 37°C under anaerobic conditions in BHI supplemented with hemin, K_2_HPO_4_, vitamin K1, and L-cysteine. The commensal *Escherichia coli* strain DH5α (Tiangen, China) was cultured in Luria-Bertani (LB) medium overnight at 37°C in shake cultivation at 220 rpm/min.

**4 Neutrophil isolation**

Fresh mouse intestinal tissue was treated to generate a single-cell suspension using Liberase (Roche, Switzerland) and DNase I (Roche, Switzerland). The NEs were isolated by gradient centrifugation using different ratios of Percoll medium (Sigma-Aldrich, USA).

**5 Neutrophil migration assay**

The purified NEs (2.5×10^5^/well) were introduced into the upper chamber of the Transwell system (8.0 μm pore size; Falcon), while *F. nucleatum* (1.0×10^8^ CFU/well) and Caco-2 cells (1.0×10^6^/well) were introduced into the lower chamber. After pre-defined time periods, the upper chamber was detached, and the cells were fixed using methanol. Subsequently, the cells on the membrane were stained with a crystal violet solution, photographed, and quantified.

**6 Gelatin zymography**

To measure the activity of MMPs, conditioned medium and anastomotic tissues were prepared as follows: Conditioned media in all samples was adjusted to ensure that all had the same protein concentration. Then, 5× non-reducing sample buffer was added to the samples. Gels (7.5% acrylamide containing gelatin) were prepared using 1 mm thickness plates, and samples were loaded into each well. A protein molecular weight marker was loaded in one well in each gel. The gels were run at 150V until good band separation was achieved. The gels were then washed twice for 30 minutes with washing buffer to remove SDS, followed by rinsing for 5–10 minutes in incubation buffer at 37°C with agitation. The buffer was removed, and fresh incubation buffer was added, and samples were incubated for 24 h at 37°C. The incubation buffer contains cofactors necessary for the gelatinase reaction to occur. After the incubation, the gels were incubated with staining solution for 30 minutes, then with destaining solution until bands could be clearly seen. Areas of enzyme activity appeared as white bands against a dark blue background.

**7** **Flow cytometry**

The isolated NEs were washed with flow cytometry staining buffer to dissociate them into single cells. To identify NEs, the cells were stained with FITC-labeled anti-Ly6G monoclonal antibody (BioLegend, USA), PE-labeled anti-Ly6C monoclonal antibody (BioLegend, USA), and APC-labeled anti-CD11b monoclonal antibody (BioLegend, USA). After 15 minutes of staining, the cells were washed twice with staining buffer. After washing, the percentage of Ly6G^+^ cells was analyzed immediately using flow cytometry.

**8 H&E and Masson trichrome staining**

The tissue sections from the anastomosis were washed, dehydrated, and embedded in paraffin after being fixed in 4% paraformaldehyde for 48 h. The specimens were sectioned at 4 μm thickness using a microtome. Before staining, paraffin sections were dewaxed with xylene, rehydrated in graded ethanol, and then stained according to the instructions provided with the HE Staining Kit and Masson’s Trichrome Stain Kit. After they were dehydrated with xylene, the slides were preserved with a neutral resin.

**9 Immunohistochemical staining**

Immunohistochemistry (IHC) for collagen Ⅰ, collagen Ⅲ, MPO, Ly6G, MMP9, MMP2, NOD1, RIPK2, and p-ERK was performed using colon sections obtained from the mouse model of anastomotic leakage. After dewaxing, rehydration, and being washed in PBS, the sections were retrieved in 0.5M EDTA buffer (pH 8.0) (Solarbio, China). The following steps were conducted using an Ultrasensitive SP kit, based on the manufacturer’s protocol (Maxim Biotechnologies, China): Cells were incubated with antibodies against MPO and Ly6G at four degrees overnight. The following day, the sections were photographed under a light microscope at proper magnifications.

**10 Fluorescence *in situ* hybridization (FISH)**

A Cy3-conjugated *F. nucleatum* 16S rRNA probe (5’-CTT GTA GTT CCG C(C/T) TAC CTC-3’) was used for the FISH assay to detect the existence of *F. nucleatum* in samples ^10^. The dewaxed and rehydrated paraffin tissue sections were treated with proteinase K and fixed with 1% paraformaldehyde, then incubated with the pro-hybridized buffer for 3 h at 37°C. The sections were incubated with a mixture of hybridization buffer and the probe for 18h in a dark chamber at 42°C. After sections were counterstained with DAPI, the images were captured with a fluorescence microscope.

**11 Multiple immunofluorescence labeling**

The sections were treated with 3% hydrogen peroxide and Tris-EDTA buffer (pH 9.0), permeabilized with 1% Triton X-100 and blocked with 5% BSA for 1 h, then incubated with primary antibodies against MMP9 and Ly6G overnight at 4°C. After they were incubated with a fluorescent secondary antibody, the slides were counterstained with DAPI, and images were captured with a fluorescence microscope.

**12 Transcriptome sequencing and bioinformatic analysis**

Caco-2 cells from the logarithmic growth phase were inoculated into a 6-well plate, with 1×10^6^ cells per well, and cultured for 24 h. The indicated amount of *F. nucleatum* was suspended in DMEM and co-cultured with the Caco-2 cells at a ratio of 100:1 for 4 h to generate the infection model. Total RNA was isolated using Trizol reagent (Invitrogen). The RNA-seq transcriptome library was prepared using the TruSeq™ RNA sample preparation kit from Illumina (San Diego, CA), following the manufacturer’s instructions, using 1 μg of total RNA. The library was then quantified using TBS380, and a paired-end RNA-seq sequencing library was created and sequenced with the Illumina HiSeqxten/NovaSeq 6000 sequencer (2×150bp read length). Differential expression analyses were performed using DESeq2 or DEGseq. DEGs with |log2FC|≧1 and FDR < 0.05 (DESeq2) or FDR < 0.001(DEG) were considered to be significantly different expressed gene. In addition, functional-enrichment analysis including Gene Ontology (GO) and Kyoto Encyclopedia of Genes and Genomes (KEGG) were performed to identify which DEGs were significantly enriched in GO terms and metabolic pathways at Bonferroni-corrected P-value <0.05 compared with the whole-transcriptome background. GO functional enrichment and KEGG pathway analysis were carried out by Goatools and Python scipy software, respectively.

**13 RT-PCR**

Total RNA was isolated using the Trizol reagent (Invitrogen), and 1 μg of total RNA was reverse transcribed using the ReverTra Ace® qPCR RT Master Mix with gDNA Remover (Toyobo, Japan). Real-time quantitative PCR (qPCR) was performed using a Genomic DNA Purification Kit (Tiangen, China). The Ct values obtained from different samples were compared using the 2^−ΔΔCt^ method. Glyceraldehyde-3-phosphate dehydrogenase (GAPDH) served as an internal reference transcript. The primers used are listed in **Table 1**.

**14 Western blotting (WB)**

Total cellular protein was isolated from cultured cells and mouse colons using a protein extraction solution (Beyotime, China). The proteins were separated by 10% and 12% sodium dodecyl sulfate-polyacrylamide gel electrophoresis (SDS-PAGE) and transferred to polyvinylidene fluoride (PVDF) membranes at 300 mA for two hours at four degrees, utilizing a wet-blotting apparatus (Bio-Rad). The membranes were blocked with 5% non-fat milk for an hour at room temperature and then incubated overnight at four degrees with primary antibodies diluted in TBST. Subsequently, the membranes were incubated with appropriate secondary antibodies for 1 h at room temperature. Protein signals were detected using the ChemiDocTM XRS+ system (Bio-Rad). The following primary antibodies were used for WB: MMP9 (10375-2-AP, Proteintech), MMP2 (10373-2-AP, Proteintech), MPO (22225-1-AP, Proteintech), Ly6G (ab238132, Abcam), cit-H3 (ab219407, Abcam), neutrophil elastase (NE) (ab131260, Abcam), NOD1(sc-398696, Santa Cruz), RIPK2 (sc-166765, Santa Cruz), ERK1/2 (11257-1-AP, Proteintech), p-ERK1/2 (28773-1-AP, Proteintech), and β-actin (66009-1-Ig, Proteintech).

**15 ELISA**

The supernatant of Caco-2 cells cultured for 4 hours with or without *F. nucleatum* was collected, and the concentrations of inflammatory cytokines (IL-1β, IL-2, IL-3, IL-6, IL-8, IL-12, and IL-23) were measured using Elisa kits (Elabscience, Wuhan, China) according to the manufacturer’s instructions. A microplate reader (Thermo Fisher Scientific. Inc. MA, USA) was used to detect the absorbance values at the wavelength of 450 nm.

**16 Statistical analysis**

Normally distributed data were analyzed using an independent sample t-test, and data with a skewed distribution were analyzed by the Mann-Whitney U test. The Pearson chi-squared test was used to compare categorical data. Bioinformatic analyses were carried out using the R software program (www.r-project.org). Statistical analysis was performed using Prism 9.5 software, and data from at least three independent experiments are expressed as the mean ± standard error (SE). Error bars in the scatterplots and bar graphs represent the SE. Differences were considered significant when **p* < 0.05, ***p* < 0.01, or ****p* < 0.001.
